# Vehicle Unpaved Road Response Spectrum Acquisition Based on Accelerometer and GPS Data

**DOI:** 10.3390/s120809951

**Published:** 2012-07-25

**Authors:** Nan Cong, Jianzhong Shang, Yanxi Ren, Yao Guo

**Affiliations:** 1 College of Mechanical Engineering and Automation, National University of Defense Technology, Changsha, Hunan 410073, China; E-Mails: sjz1966@hotmail.com (J.S.); guoyao.cn@gmail.com (Y.G.); 2 System Institute of Engineering Equipment, Beijing 100093, China; E-Mail: renyanxi@yahoo.com

**Keywords:** road response spectrum, road simulation, GPS, durability test, sensor fusion

## Abstract

This paper describes a response acquisition system composed of some spindle accelerometers and a time synchronized on-board GPS receiver developed in order to collect the dynamic response of vehicle riding on an unpaved road. A method of time-space conversion for calculating the response spectrum is proposed to eliminate the adverse effect of time-varying speed, based on the transform from the equitime sampled spindle acceleration responses to equidistance sampling. By using two groups of independent distance histories acquired from GPS, a method called long-range error correction is proposed to improve the accuracy of the vehicle's distance information, which is critical for the time-space conversion. The accuracy and limitations of the system have been analyzed, and its validity has been verified by implementing the system on a wheel loader for road response spectrum measuring. This paper offers a practical approach to obtaining unpaved road response spectra for durability road simulation.

## 1. Introduction

The unpaved road simulation (RS) is an essential element of the durability tests for off-highway vehicles. As the simulation input, the road roughness profile and its accuracy have a direct effect on the test's credibility. Lots of paved road profile models have already been built up and can be automatically generated by most simulation control systems [1]. However, with regard to the unpaved roads (or even terrains), there're no standardized road spectra to utilize because of the potential complexity and specified aim-leading development of the road situation. Therefore, unlike the paved road simulation, an *in situ* acquisition operation is always needed before performing any unpaved road simulation.

Since the 1960s a lot of studies had been done for measuring paved road profiles [2], and many kinds of measuring systems had been invented [3–6]. Nowadays, the most commonly used non-contact profilometers can measure the road spectra with pretty high accuracy [7–9]. Nonetheless, this delicate system may not yield useful result for the unpaved road simulation due to the following reasons:

1. The ordinate values of the road profile mainly depend on the data provided by some sort of relative displacement sensor (laser, infrared or ultrasonic) mounted on the chassis of the host vehicle. The obtained final displacement (after deducting the chassis fluctuations) could represent the road input only when the road surface itself is not excessively interrupted by the vehicle riding though it. Obviously, this hypothesis is not true for unpaved roads due to the large-scale road surface settlement of the unpaved road.
2. The abscissa values of the road profile mainly depend on the vehicle speed/distance information measured by an odometer or a rotational velocity sensor mounted on the spindle of the host vehicle. However, this speed/distance information could contain large errors if there's a major variation in the vehicle speed or a high level of wheel slip ratio which often occurs during unpaved road travel [10,11].

Fortunately, the “real” road profile is not necessary for RS. Based on the fact that the durability test result always depends on loads acting on the vehicle's wheel axles, it is reasonable to collect the spindle responses (also called the “effective” road profile) instead of road spectra as the input of RS [12], which can easily avoid the first disadvantage mentioned above. Actually, there is a category of road measurement equipment called the Response Type Road Roughness Measurement System (RTRRMS) which calculates the road profile from the acquired vehicle axle responses. This category of equipment is not popular today for road measuring due to the fact its output data is dependent on the host vehicle, which results in two unwanted effects—it is untransportable (which means the results measured on one vehicle cannot be used for others) and unstable with time [13,14]. Considering that the *in situ* acquisition operation is originally conducted for one specific type of off-highway vehicle, the untransportability of acquired responses is not a problem anymore for our research. The reason for the time unstability is similar to the second problem listed above.

To solve the second problem, a device is need to measure the vehicle's speed/distance information accurately and irrelevant to the road conditions and driving state. The best way to fulfill that is using a Global Positioning System (GPS) receiver supplemented with Real Time Kinematics (RTK) technology. The RTK-GPS receiver can easily measure the distance of vehicle with centimeter level accuracy and has been widely used in road surveying applications [15,16]. However, both the money and time cost to constructs RTK-GPS (virtual) reference station is relatively too high to implement in frequent *in situ* data acquisition operations. Additionally, the acquired unpaved roads are typically in the areas where the RTK service is not available (especially in China [17]).

This paper proposes a vehicle response acquisition system oriented to the unpaved road simulation. The objectives of this paper were to develop an easily implemented measuring system based on the spindle acceleration response and time-synchronized GPS distance information which only uses economical accelerometers and GPS receivers without RTK. To improve the accuracy of the distance measured by the ordinary GPS receiver, a calibration method called long-range error correction (LEC) is discussed in detail. The limitation and accuracy of the system was analyzed as well. The proposed acquisition system has been applied in an unpaved proving ground RS measurement of a wheel loader, and comparison works have been done to evaluate the validity of GPS displacement calibrations and BRS time-space conversion methods presented in this paper.

## 2. Response Acquisition System

**Figure 1.**
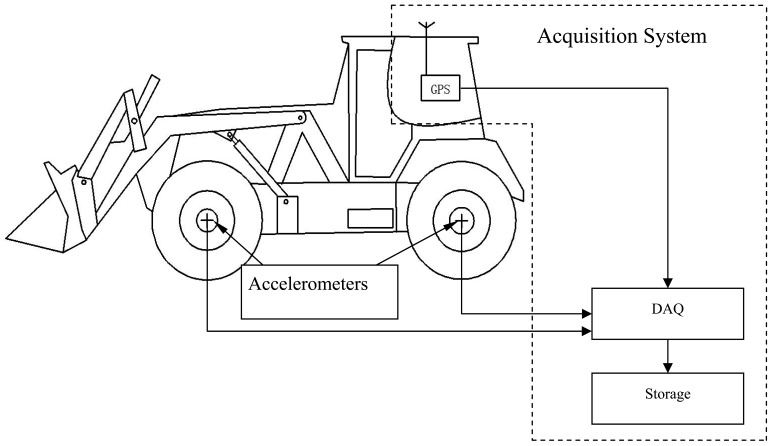
Vehicle response acquisition system.

As shown in Figure 1, the acquisition system consists of four groups of Endevco-7596 accelerometers on each spindle of the host vehicle as well as a set of data collection systems with an ordinary GPS module. During the operation, the sensors simultaneously record the acceleration histories *a*(*t*) at all axles, longitude and latitude history *λ*(*τ*), *Ø*(*τ*) and speed history *v*(*τ*) of the vehicle. Here, *t* and *τ* all indicate the time, while they stand for possibly different time sampling intervals of the acceleration and GPS output histories. The sampling frequencies corresponding to acceleration and GPS data are *F_sa_* and *F_sGPS_*.

Generally, the response frequency of accelerometer is high enough for the acquisition. The bandwidth requirement for the durability test is almost the lowest comparing with the rest kinds of road simulation, however, the cutoff frequency *F_H_* is critical for the acquisition system due to the constrained GPS output frequency *F_sGPS_*. Deduced from the road spatial wavelength resolution calculation equations [13] and Nyquist sampling theorem, the *F_sGPS_* should be at least two times higher than the *F_H_*. In this paper, the cutoff frequency *F_H_* = 30 Hz and the output frequency of GPS need to be at least higher than 60 Hz. That is much higher than possible with an ordinary commercial GPS receiver (usually only 1∼10 Hz), so a high output frequency GPS receiver developed from a field programmable gate array (FPGA) with the *F_sGPS_* = 100 Hz is used here to implement the acquisition operation.

## 3. Time-Space Conversion of Response History

According to the principle of GPS [18], if vehicle steering and elevation variation are not considered, two kinds of vehicle travel distance histories can be calculated independently by the longitude/latitude histories *λ*(*τ*)/*Ø*(*τ*), and speed history *v*(*τ*). The distance history that obtained by speed integration can be called the integral distance history *S*_1_(*τ*):

(1) $$S_{1}(i){|}_{i=2,3,\ldots ,\tau }=S_{1}(i-1)+v(i-1)\Delta \tau$$

where Δ*τ* = *1/F_sGPS_* denotes the GPS sampling interval.

The distance history that obtained by Haversine formula [19] from longitude/latitude histories *λ*(*τ*)/*Ø*(*τ*) can be called the positioning distance history *S*_2_(*τ*):

(2) $$S_{2}{\left(i\right)}_{i=1,2,\ldots ,\tau }=2r\arcsin\sqrt{\sin^{2}\left(\frac{\phi (i)-\phi_{0}}{2}\right)+\cos\phi_{0}\cos\phi (i)\sin^{2}\left(\frac{\lambda (i)-\lambda_{0}}{2}\right)}$$

where *r* ≈ 6,371.01 km indicates the average radius of the earth; the *λ*_0_ and *Ø*_0_ indicate the initial longitude and latitude of the vehicle. Besides, for further improving the accuracy of this positioning distance history, the more accurate “Vincenty formula” [20] could also be used to calculate the distance history. The Vincenty formula is a kind of iterative algorithm which can achieve an accuracy at about 0.6 mm for every calculation point.

Theoretically, the *S*_1_(*τ*) and *S*_2_(*τ*) should be identical, and any of them could be used to combine with the acceleration history *a*(*t*) to make the space-domain history *a*(*S*). Then, the power spectral density (PSD) of acceleration *G_a_*(*n*) which is critical for the road simulation can be calculated, where *n* denotes the space frequency, in m^−1^.

**Figure 2.**
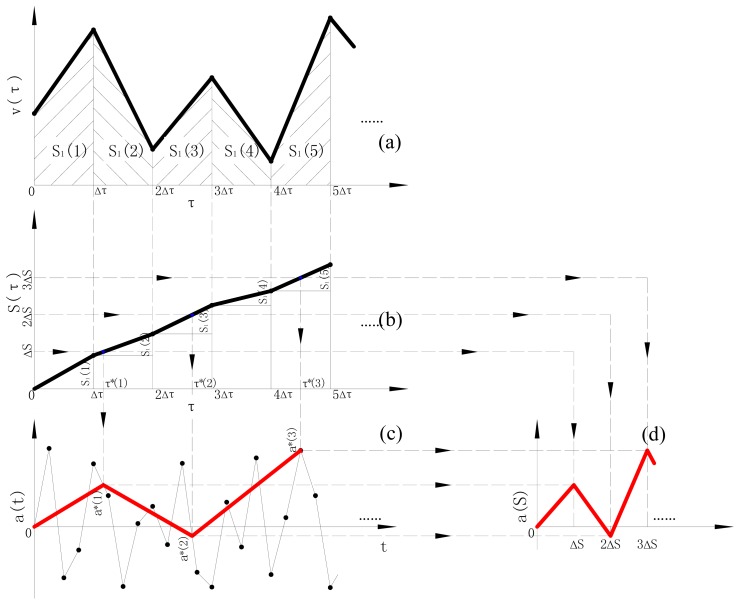
Acceleration history time-space conversion.

As mentioned previously, because of the different sampling frequencies the accelerometer and GPS receiver possess, the acceleration history *a*(*t*) and distance history *S*(*τ*) are not in point-to-point correspondence. Therefore, a sensor fusion procedure is needed. The procedure converts the equitime acquired acceleration history *a*(*t*) to an equispace acceleration history *a*(*S*) by two interpolation operations, as shown in Figure 2 (taking *S*_1_(*τ*) as example):

1. As illustrated in Equation (1), the integral distance history *S*_1_(*τ*) is obtained by integration from the speed history *v*(*τ*), as shown in Figure 2(a,b);
2. Based on the cutoff frequency *F_H_* and the average speed *v̄*, it estimates the required minimum space sampling interval Δ*S* ≤ *v̄*/2*F_H_* and gets the corresponding interpolated time τ*(*i*)|_*i*=*1,2,…*_ on each even Δ*S* through the whole distance history *S*_1_(*τ*), as shown in Figure 2(b);
3. Based on the acquired acceleration history *a*(*t*), interpolated acceleration amplitudes a*(τ*) corresponding to each τ*(*i*)|_*i*=*1,2,…*_ are obtained, as shown in Figure 2(b,c);
4. Plot the interpolated acceleration a*(τ*) on the space coordinates with the sampling interval of Δ*S*, forming the acceleration history *a*(*S*), as shown in Figure 2(b–d).

In some sense, the newly combined space-domain acceleration history *a*(*S*) is a kind of road profile and the vehicle speed is irrelevant. So, when the vehicle speed is time-varying as shown in Figure 2(a), it could be used to calculate the space-domain acceleration spectrum *G_a_*(*n*) through Fast Fourier Transform (FFT) based on the *a*(*S*), more accurately than the calculation directly using the *a*(*t*) and its PSD *G_a_*(*f*) by the following Equation:

(3) $$G_{a}(\bar{n})=\frac{G_{a}(f)}{{\bar{v}}^{3}}$$

Where *n̄* = *f/v̄* denotes the averaged space frequency, in m^−1^.

It can be concluded from Figure 2 that, the distance histories have a significant impact on the accuracy of the interpolated space history *a*(*S*) and its spectrum *G_a_*(*n*). Actually, the noises contained in the histories of speed and geographic data measured by the GPS can produce large errors in the distance history *S_i_*(*τ*)|_*i*=*1,2*_ regardless of which equation, Equation (1) or Equation (2), is used.

To improve the accuracy that is used in the time-space conversion procedure, we will discuss a calibration method in the next section based on the characteristics of the errors in the two kinds of distance histories.

## 4. Distance History Calibration and Accuracy Analysis

The error contained in the GPS measured vehicle speed *v*(*τ*) can be assumed as a Gaussian noise with a bias *δ̄_GPS-v_* and standard deviation σ*_GPS-v_*, with the unit of m/s. With regard to a certain vehicle speed *V*, the short-range absolute accuracy of *S*_1_(*τ*) in one sampling interval is:

(4) $$\delta_{S-S_{1}}\leq \frac{({\bar{\delta }}_{\text{GPS}-v}+3\sigma_{\text{GPS}-v})\Delta S}{V}$$

Affected by the integral cumulative error, the long-range absolute accuracy of *S*_1_(*τ*) in a road with length *L* is:

(5) $$\delta_{L-S_{1}}\leq \frac{{\bar{\delta }}_{\text{GPS}-v}L}{V}$$

As for the positioning calculated distance history *S*_2_(*τ*), its absolute accuracy is only related to the positioning error of GPS receiver but irrelevant to the length of road, which can be estimated by:

(6) $$\delta_{S_{2}}\leq 2{\text{CEP}}_{\text{GPS}-p}$$

where CEP*_GPS-p_* denotes the positioning Circular Error Probability (CEP) of GPS receiver, with the unit of m.

**Figure 3.**
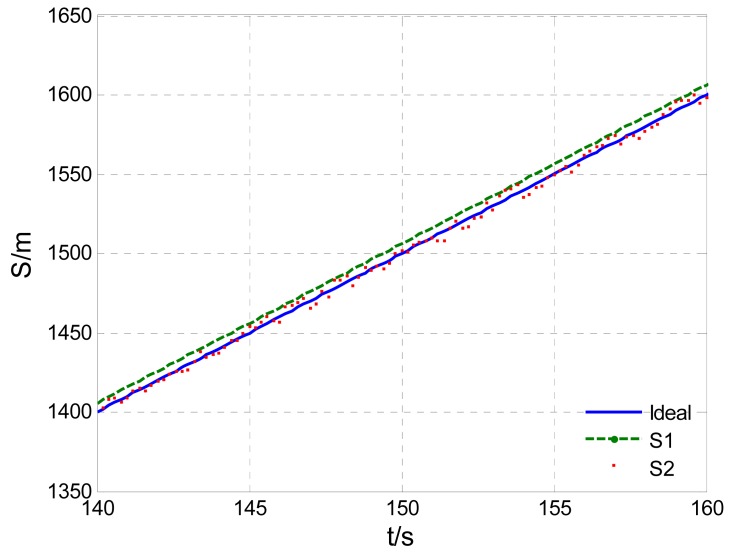
GPS calculated distance histories *vs.* theoretical distance history.

**Figure 4.**
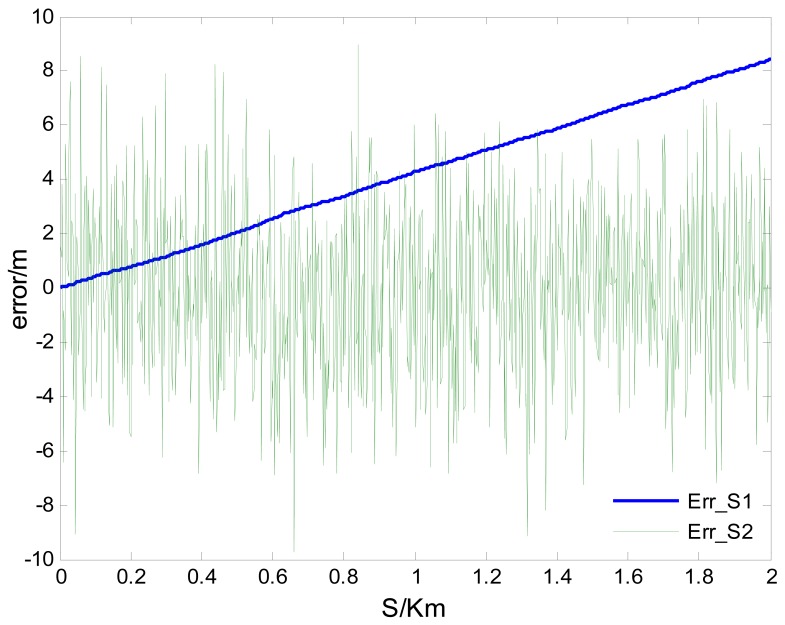
GPS calculated distance errors varies with travel length increasing.

Supposing *V*=10 m/s, *δ̄_GPS-v_* = 0.04 m/s, σ*_GPS-v_* = 0.03 m/s and CEP*_GPS-p_* = 3 m, the simulated velocity integral travel history *S*_1_ and positioning calculated distance history *S*_2_ in comparison with the theoretical distance history, are shown in Figure 3. Their distance errors vary with travel length as shown in Figure 4.

Illustrated by Figures 3 and 4, the characters of those two GPS calculated distance histories are as follows: the integral distance history *S*_1_ has higher accuracy (for the receiver used in the paper, the root mean square speed measurement error RMS*_GPS-v_* < 0.05 m/s) at the beginning of acquisition. However, due to the accumulated integration error impact, as the distance increases, the precision of *S*_1_ will reduce. On the contrary, the positioning calculated distance history *S*_2_ is of low absolutely accuracy during the whole trip (95% CEP*_GPS-p_* < 3 m, as in this paper). Its error level will not change with increasing distance. Namely, the relative positioning error to travel distance will decrease with the increasing travel distance. As shown in Figure 4, there exists a critical length *S_L_*. When the acquisition length is shorter than the *S_L_, S*_1_ will be more accurate. Conversely, if the acquisition length is longer than *S_L_, S*_2_ will be more applicable. The critical length *S_L_* can be deduced by Equations (5), (6) with the Equation below:

(7) $$S_{L}=\frac{2{\text{CEP}}_{\text{GPS}-p}V}{{\bar{\delta }}_{\text{GPS}-v}}$$

As shown in Equation (7), the critical length *S_L_* is decided by the vehicle speed *V* and the ratio of GPS positioning accuracy CEP*_GPS-p_* to the speed-measuring bias *δ̄_GPS-v_*.

Because of the local (short-range) accuracy is more important for the time-space conversion, *S*_1_ is always prior to be chosen as the base history. When the data acquisition length is longer than *S_L_*, a method called long-range error correction (LEC) can be applied to improve the long-range accuracy of *S*_1_. The correction steps are shown as follows:

1. Select a correction length *S_M_*, making sure *S_M_* ≥ *S_L_*;
2. The Equations (1),(2) are used to calculate the short-range integral history *S*_1_(*i*)|_*i*=*1,2,…l*_ and positioning calculated history *S*_2_(*i*)|_*i*=*1,2,…l*_ till *S*_2_(*l*) ≥ *S_M_*;
3. Comparing *S*_1_(*l*) and *S*_2_(*l*) and defining the long-range error *e* = *S*_1_(*l*) − *S*_2_(*l*);
4. Correcting the integral history *S*_1_(*i*)|_*i*=*1,2,…l*_ to make the new refined history:
  (8) $$S_{1}^{*}{\left(i\right)}_{i=1,2,\ldots l}=S_{1}(i)-\frac{i-1}{l-1}e$$

The *S*_1_* has the same length as that of the *S_M_*, but with improved accuracy both in short-range and in long-range which are shown as follows separately:

(9) $$\delta_{S-S_{1}^{*}}\leq \frac{2{\text{CEP}}_{\text{GPS}-p}\Delta S}{S_{M}}+\frac{3\sigma_{\text{GPS}-v}\Delta S}{V}$$

(10) $$\begin{array}{cc} \delta_{L-S_{1}^{*}}\leq \frac{2{\text{CEP}}_{\text{GPS}-p}l_{M}}{S_{M}} & \left(0<l_{M}\leq S_{M}\right) \end{array}$$

Defining the local accuracy as the ratio of short-range accuracy to the sampling interval: *ε* = *δ_S_*/Δ*S* (generally, 0 < *ε* ≤ 10%), the local accuracy of corrected history can be obtained by Equation (9):

(11) $$\varepsilon \geq \frac{\max\delta_{S-S_{1}}}{\Delta S}=\frac{2{\text{CEP}}_{\text{GPS}-p}}{S_{M}}+\frac{3\sigma_{\text{GPS}-v}}{V}$$

Deduced from Equation (11), the minimum correction length *S*_*Mm*in_ that satisfied with the local accuracy requirement *ε*_0_, is:

(12) $$S_{M\min}=\frac{2{\text{CEP}}_{\text{GPS}-p}V}{\varepsilon_{0}V-3\sigma_{\text{GPS}-v}}$$

It can be deduced from Equations (7), (9), (10) and (12) that, when conducting the acquisition operation with a given GPS receiver, for obtaining a corrected distance history both with local and long-range accuracy improvements, the correction length and vehicle velocity should meet the conditions as follows:

(13) $$S_{M}\geq \max(S_{M\min},S_{L})$$

(14) $$V>\frac{3\sigma_{\text{GPS}-v}}{\varepsilon }$$

**Figure 5.**
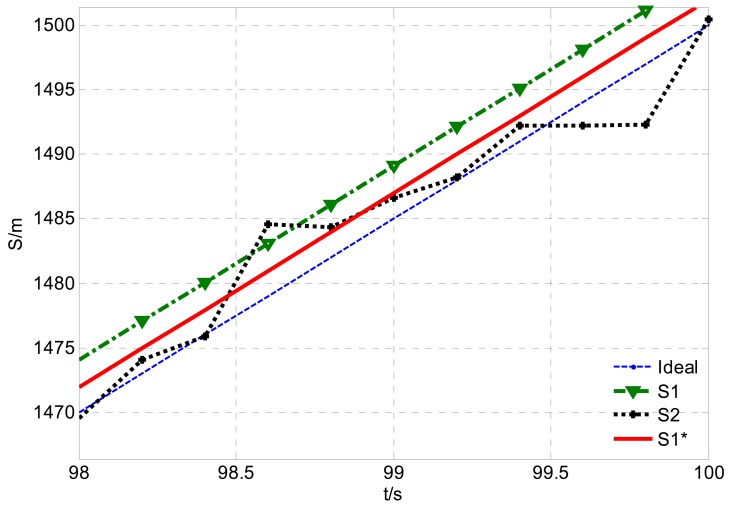
Distance histories before and after LEC *vs.* theoretical distance history.

**Figure 6.**
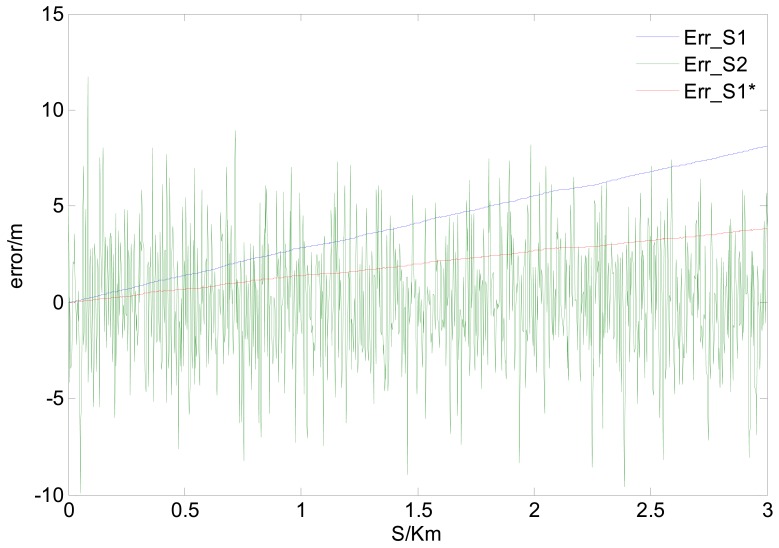
GPS calculated distance errors before and after LEC varies with increasing travel length.

To demonstrate the effect of LEC, under the local accuracy requirement of *ε*_0_ = 1%, we continue to use the parameters of GPS measurement errors as same as those in Figures 3 and 4. The correction length *S_M_* and vehicle speed *V* are set to 3 km and 15 m/s separately, to satisfy the expression shown in Equations (13), (14). The distance histories before and after LEC comparing with the theoretical distance history are shown in Figure 5, and their distance errors varying with travel length are shown in Figure 6.

As shown in Figures 5 and 6, the travel distance error can be significantly reduced after LEC. The short-range and long-range accuracy can be calculated by Equations (9), (10), with the result of 1.2 mm and 2 m/km (it can be further reduced by moving average processing), respectively.

It should be noted that though the GPS positioning and velocity-measuring accuracy can be affected by lots of external conditions, e.g., the weather conditions, the number of satellites in view and geometry, the dynamic characteristics of host vehicles, *etc.*, the equations in this section always product reasonable results to evaluate the acquisition system because of the conservative result they produce.

## 5. Application

**Figure 7.**
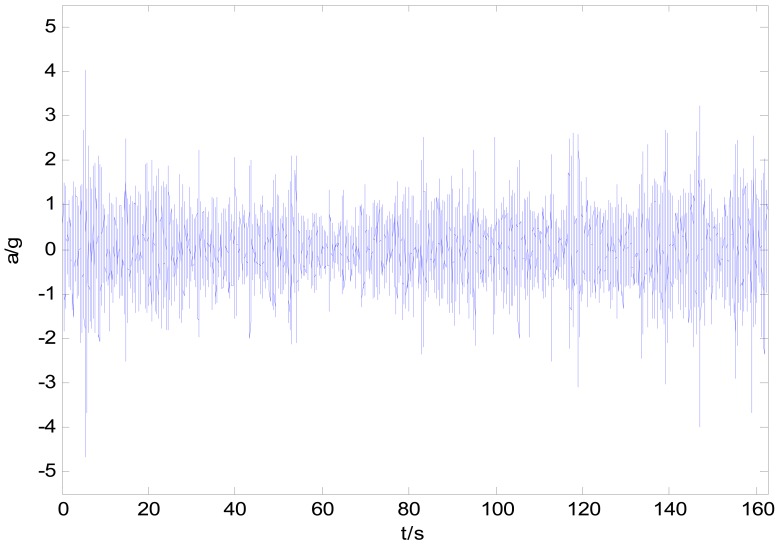
Original acceleration response time history at left front wheel axles.

**Figure 8.**
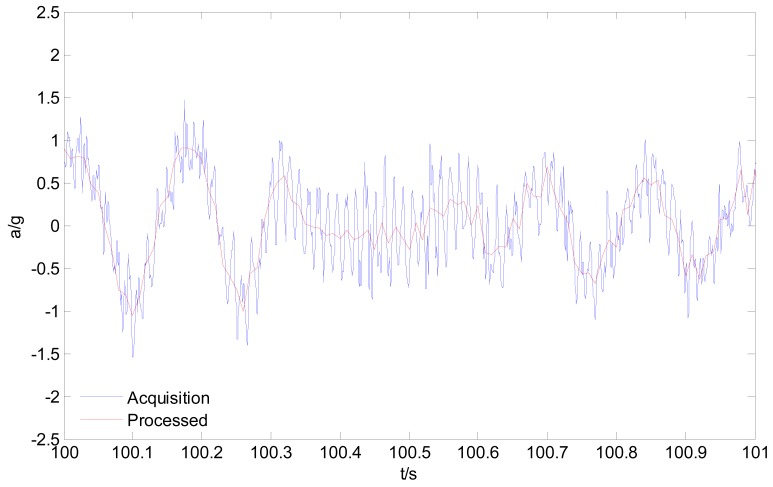
Acceleration response histories before and after resampling.

By using the acquisition system shown in Figure 1, all four wheels' acceleration responses (with frequency of 2 kHz) had been acquired during a wheel loader riding through a gradeless straight unpaved proving ground (PG) road. Considering that the low vibration frequency band (<30 Hz, as above mentioned) is useful for the durability tests, an anti-aliasing low-pass filter and resampling process with frequency of 100 Hz are implemented to the original collected signal. Figure 7 gives the time response history of the left front wheel. Figure 8 presents the comparison result of the processed wheel response with original acquired signal in one second.

**Figure 9.**
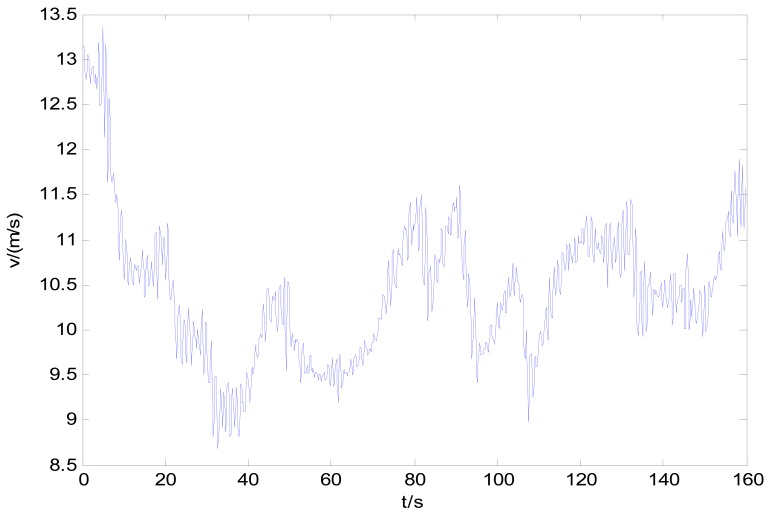
Vehicle speed history.

Figure 9 shows the vehicle speed history of the acquisition operation, which indicates that the speed can't be effectively stabilized due to the PG's poor road condition. The vehicle speed varies significantly during the whole trajectory.

**Figure 10.**
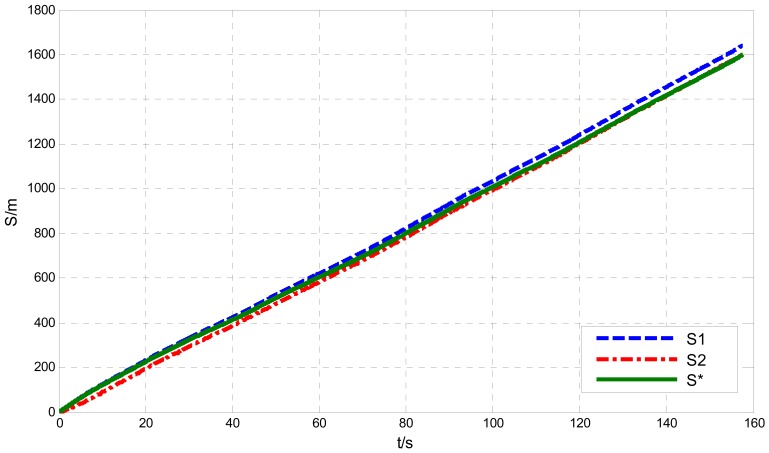
Distance histories before and after LEC.

During the acquisition operation, the positioning accuracy is almost as same as it declared. However the speed-measuring accuracy of the GPS receiver at field is a little worse than labeled at about 0.05 m/s for both the bias and standard deviation. Substituting those parameters along with the mean speed calculated from the speed history shown in Figure 9 (10.43 m/s) into Equations (7), (12), (13), it can be concluded that the correction length should be no shorter than 1,252 m. Because of the acquisition road length is longer than the minimum correction length, we use it as correction length for the LEC. Figure 10 gives the whole-journey speed integral distance *S_1_*, positioning calculated distance *S_2_* and the calibrated distance history S*. The result shows that, the calibrated history coincides with the speed integral history at first, and approaches the positioning calculated history gradually as the travel distance increases. Known from section 3 of the paper, the corrected history S* will keep a reasonable accuracy of both the total travel length and the adjacent data.

**Figure 11.**
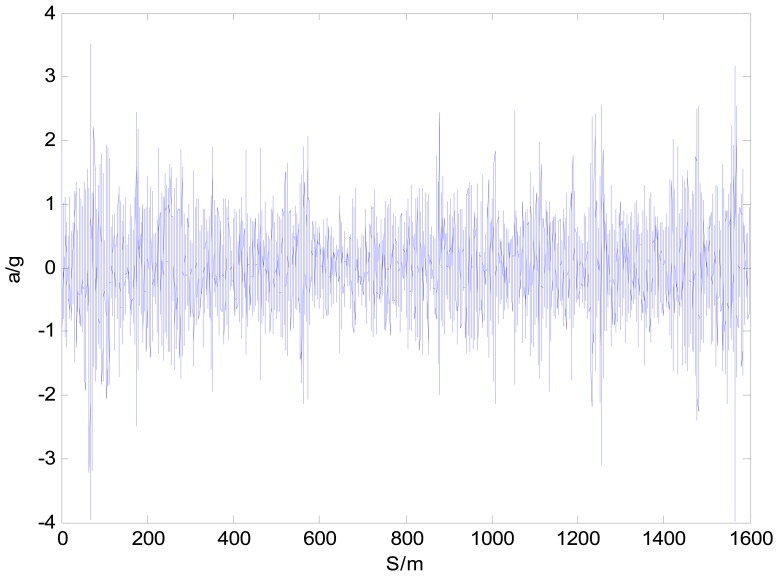
Acceleration response space history of the front wheel axle.

**Figure 12.**
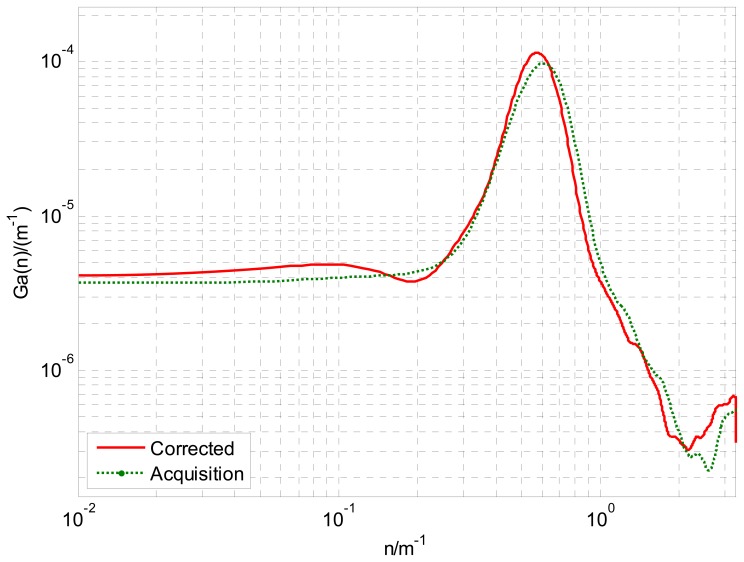
Space PSD of acceleration *G_a_*(*n*).

**Figure 13.**
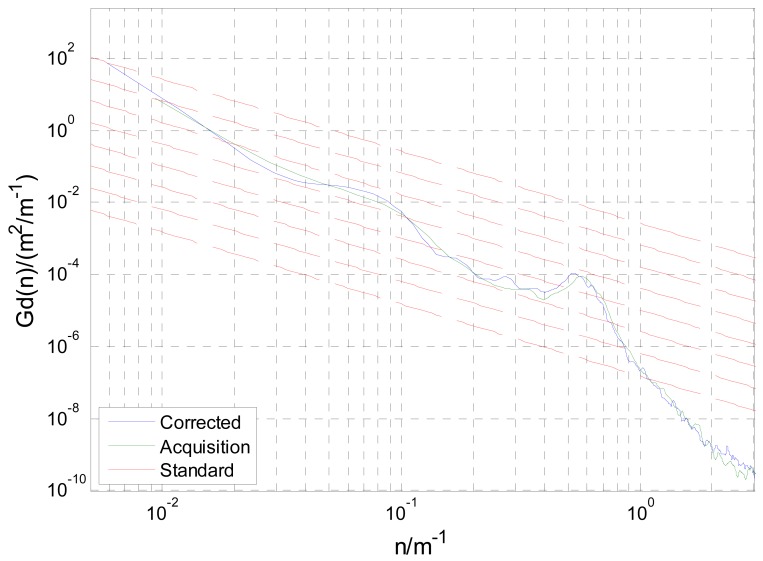
Space PSD of displacement *G_d_*(*n*).

The equi-distance sampled acceleration history is shown in Figure 11. For comparison, when the simulation speed is set to the mean speed of acquisition, by the method raised in this paper and that directly obtained through Equation (3), the road's space acceleration spectrum *G_a_*(*n*) and displacement spectrum *G_d_*(*n*) are plotted in Figures 12 and 13 respectively. In Figure 13, eight standard road spectra according to ISO8068 have been plotted as references.

It can be concluded from Figures 12 and 13 that, in the whole interested frequency range, the method raised in the paper shows its advantage by obtaining a more accurate PSD as taking the vehicle speed variation into consideration. The proposed method can adapt speed variation on a much larger scale that shown in Figure 9, and produces accurate road response spectra for road simulation. Furthermore, with different space interval Δ*S* and supposed vehicle speed *V*, the acquisition system and calibration method can be used for processing a wide range of unpaved road.

## 6. Conclusions

The acquisition system integrated by GPS and spindle accelerometers can be easily mounted and quickly deployed on a host vehicle to acquire the vehicle response for road simulation. With the velocity integral displacement history and the positioning calculated displacement history independently obtained by a high output frequency GPS receiver, the vehicle's displacement can be accurately acquired by the LEC calibration method. By using this accurate displacement history, time-space conversion could be applied for the spindle acceleration histories to acquire the response spectrum which avoids the effect of vehicle speed variation.

Though the methods proposed in this paper are derived under the gradeless straight road situation, the methods are applicable for measuring of all kinds of road after proper amendments as the GPS information includes the value of elevation and three-dimensional speed. Moreover, aided with a higher output frequency GPS receiver and more accurate devices such as the RTK-GPS *etc.*, the methods can also be applicable to some other applications such as the road surveying and Noise-Vibration-Harshness (NVH) assessment with wider bandwidth and higher accuracy requirements.
